# Dissociating stimulus and task effects on neural oscillations during n-back tasks

**DOI:** 10.3389/fnhum.2026.1883989

**Published:** 2026-09-23

**Authors:** Jaap Munneke, Elnaz Vafaei, Jahla B. Osborne, Mahsa Shalchy, Susanne M. Jaeggi, Aaron Seitz

**Affiliations:** 1 Brain Game Center for Mental Fitness and Well-Being, Northeastern University, Boston, MA, United States; 2 Department of Psychology, Northeastern University, Boston, MA, United States; 3 Department of Neurology, University of Minnesota, Minneapolis, MN, United States; 4 Department of Psychology, University of California at Riverside, Riverside, CA, United States

**Keywords:** alpha rhythm, EEG oscillations, event-related synchronization/desynchronization, n-back task, task design, theta rhythm, time-frequency analysis, working memory

## Abstract

**Introduction:**

N-back tasks are widely used to assess working memory functioning, yet different methodological approaches between tasks and across laboratories can yield distinct neural signatures. While working memory-related event-related potentials (ERPs) have been extensively studied, it remains unclear how task parameters influence the neural oscillatory activity underlying working memory functioning.

**Methods:**

We conducted time-frequency analyses on EEG data from 36 participants performing nine different variants of the n-back task that systematically varied in stimulus type (words, pictures, colors) and task structure (timing, response, and feedback parameters). We examined power modulations in the theta (4–8 Hz), alpha (8–12 Hz), and beta (12–30 Hz) frequency bands to determine which task parameters drive changes in specific neural oscillations.

**Results:**

Results revealed a functional double dissociation: frontal theta event-related synchronization was selectively sensitive to stimulus type, with ‘pictures’ eliciting greater theta power than words and colors in the task variant with the shortest total trial duration and a requirement to respond to all stimuli, but not in other task variants. In contrast, centro-parietal alpha event-related desynchronization was driven predominantly by task structure rather than stimulus content, showing reduced alpha suppression in the task variant characterized by the longest inter-trial interval and immediate accuracy feedback. Beta oscillations showed no systematic modulation at the Region of Interest level. Cluster-based permutation analyses further confirmed the early temporal specificity of both theta and alpha effects.

**Discussion:**

We conclude that theta oscillations reflect stimulus-driven encoding demands, while alpha desynchronization tracks the structural and temporal demands of the task context (modulating the need for active neural inhibition of task-irrelevant information). These findings have direct implications for standardizing n-back protocols: stimulus type and task structure exert their influence on fundamentally different oscillatory systems and must be carefully controlled depending on which neural biomarker is targeted.

## Introduction

Working memory refers to the cognitive system that temporarily retrieves, maintains, and manipulates information to facilitate higher-order cognitive functions such as learning, reasoning, problem-solving, and decision-making (Baddeley, 2012). Numerous studies have investigated working memory capacity using a range of experimental paradigms (for a broad overview, see Cowan, 2017). Among these paradigms, *n-back* tasks are widely used to gage working memory and its underlying behavioral and neural processes and they are among the most used paradigms for investigating working memory in both research and clinical settings (Jaeggi et al., 2010). In a typical n-back task, participants monitor a sequence of stimuli and indicate whether each current item matches the one presented n-positions earlier, which requires continuous maintenance and updating of information in working memory. However, methodological differences across laboratories may affect the validity and comparability of reported findings. Researchers have adopted n-back tasks with widely varying designs, including differences in stimulus type (e.g., letters, spatial locations, pictures, faces, objects), stimulus modality (e.g., visual, auditory, tactile, or dual-modality presentations), timing parameters (e.g., stimulus duration and inter-stimulus intervals), response requirements (target-only vs. two-alternative forced choice), and feedback mechanisms such as the presence, absence, or type of performance feedback (e.g., Appelgren et al., 2014; Jaeggi et al., 2003; Mohammed et al., 2017; Owen et al., 2005; Schneiders et al., 2011).

Prior work by Jaeggi and colleagues has highlighted how methodological differences in n-back tasks have contributed to inconsistent conclusions about exactly which cognitive constructs and mechanisms are indexed by derived task metrics and how results should be interpreted (Jaeggi et al., 2010; Pergher et al., 2020). Moreover, while the n-back task has demonstrated its value in clinical populations such as ADHD (e.g., Salmi et al., 2020), traumatic brain injury (e.g., McAllister et al., 1999), and dementia (e.g., Fraga et al., 2018), its use as a standardized assessment tool is limited by uncertainty about which task features affect underlying neural responses (Redick and Lindsey, 2013). Furthermore, task modifications such as introducing gamification features, altering stimulus complexity (e.g., Eng et al., 2005), or using multimodal stimulus presentation can have complex and sometimes unpredictable effects on n-back task performance (Deveau et al., 2015; Mohammed et al., 2017). The lack of systematic investigations into how specific design aspects modulate neural responses and associated behavior, makes it challenging to directly compare between studies and theoretical interpretation. As EEG provides a non-invasive measure of neural activity with precise, millisecond temporal resolution, understanding which methodological variations meaningfully affect EEG-based neural metrics can be crucial for establishing standardized protocols and ensuring that cognitive assessments yield reliable and valid biomarkers of working memory function.

Within EEG research, event-related potentials (ERPs) have been extensively used to study neural responses during n-back tasks (e.g., Dong et al., 2015; Scharinger et al., 2017; Shalchy et al., 2020). Previous studies have tentatively shown that even subtle changes in task design can lead to marked differences in ERP morphology, such as the amplitude and latency of components like the N1, P2, N2, and P3, which reflect changes in attentional allocation, working memory load, and cognitive control processes (Shalchy et al., 2020). However, ERPs capture only phase-locked neural activity that is time-locked and consistent across trials. Because ERPs are derived from averaging across trials, non-phase-locked neural activity, which may vary in timing but still reflect important cognitive processes, often averages out over trials and is therefore not embedded in the ERP averages (Ku et al., 2010). For instance, during the delay period of a working memory task, two different task structures might require vastly different amounts of top-down inhibitory control, a process indexed by centro-parietal alpha oscillations. However, because these induced rhythms are non-phase-locked (i.e., their oscillatory peaks and troughs do not align perfectly millisecond-by-millisecond across trials) traditional time-locked ERP averaging will cause such signals to cancel each other out, resulting in an identical flat baseline. In contrast, focusing on the time-frequency domain through ERD/ERS negates phase dependency by tracking total power, which can reveal differences in underlying neural resource allocation that traditional ERP waveforms mask. As such, examining the time-frequency domain through event-related desynchronization (ERD) and synchronization (ERS) is an essential additional step, as these measures capture changes in oscillatory power regardless of phase consistency, revealing task related neural patterns that complement traditional ERP analyses. Importantly, different frequency bands in the EEG spectrum may respond differentially to specific task features, potentially informing which design parameters affect distinct neural processes in working memory.

A growing body of EEG research has demonstrated that oscillatory activity within specific frequency bands is systematically modulated during working memory tasks (e.g., Palomäki et al., 2012, Tsoneva et al., 2011). Notably, increases in theta-band (4–8 Hz) power have been consistently observed over midline frontal regions and are thought to support the temporal organization and maintenance of sequentially ordered items in working memory (Cavanagh and Frank, 2014; Roux and Uhlhaas, 2014). In contrast, alpha-band (8–12 Hz) oscillations typically show task-related suppression over centro-parietal regions, which is interpreted as reflecting the active inhibition of task-irrelevant information (Jensen and Mazaheri, 2010; Klimesch et al., 2007). Beta-band neural activity (12–30 Hz) is thought to affect more general working memory processes such as focused attention and cognitive control needed to actively maintain and manipulate information, with effects distributed across central and parietal regions (Chen and Huang, 2016; Schmidt et al., 2019). Moreover, recent theoretical and empirical work suggests that cross-frequency coupling, particularly between low-frequency bands such as theta and alpha, may serve a functional role in coordinating neural processes underlying working memory (e.g., Kawasaki et al., 2010; Sauseng et al., 2009). This coupling may help coordinate different working memory processes by organizing neural activity across frequency bands, enabling the brain to handle multiple pieces of information simultaneously. Together, these findings highlight the dynamic role of neural oscillations in supporting working memory encoding, maintenance, and manipulation (Roux and Uhlhaas, 2014).

Building on previous ERP investigations during n-back task performance, which examined the influence of task parameters (e.g., stimulus type, duration, ISI, and feedback) and laboratory-specific variables (e.g., EEG hardware, room setup; Shalchy et al., 2020), we now expand the task parameter-based analysis to include neural oscillatory activity using event-related desynchronization (ERD) and synchronization (ERS) power metrics. Understanding which task parameters affect which frequency band is critical for developing standardized protocols and interpreting results across studies with different task designs. While ERP analyses provide important insights into time-locked neural responses to task-related events, they do not gage changes in brain rhythms that reflect underlying cognitive processes more broadly distributed in time. In the current study, we investigate how variations in n-back task structure (e.g., stimulus duration and inter-stimulus interval) and stimulus type (words, pictures, colors) influence oscillatory brain activity, with a particular focus on the theta, alpha, and beta frequency bands. Specifically, using an existing dataset (Shalchy et al., 2020), we analyze ERD/ERS to measure relative power changes across frontal, centro-parietal, and central electrode regions that have been associated with theta, alpha, and beta modulation during working memory tasks. We hypothesize that different frequency bands will be differentially sensitive to task parameters, predicting a functional dissociation: frontal theta will be primarily modulated by stimulus type, reflecting perceptual encoding demands, while centro-parietal alpha will be primarily modulated by task structure, reflecting broader maintenance and resource allocation demands, with beta oscillations not expected to show systematic modulation by either manipulation at our selected electrode clusters. By integrating ERD/ERS analyses, we aim to provide a more comprehensive account of how task-design features modulate oscillatory brain activity underlying working memory performance.

## Materials and methods

### Participants

Thirty-six right-handed undergraduate students (27 female, 9 male; mean age = 19.58 ± 0.97 years) were recruited from the University of California, Riverside (UCR). All participants were fluent English speakers with no self-reported psychiatric or neurological impairments. The experimental protocol was approved by the Institutional Review Board of UCR, and all participants provided informed consent prior to participation. Participants received course credit and a payment of $10 for completing two experimental sessions (see below), which lasted approximately 90 min per session (totaling approximately 180 min across both sessions). The UCR dataset used here originates from Shalchy et al. (2020).

### Experimental tasks and procedures

The current study employed nine distinct versions of the n-back task adapted from previous research conducted in our lab, varying in used stimulus type and timing parameters. As shown in Table 1, each task employed one of three stimulus types: Words (e.g., so, do, up), Pictures (e.g., apple, fish, bag), and Colors (e.g., red, green, blue). Additionally, the tasks varied in stimulus duration, inter-stimulus interval (ISI), response contingency, and feedback mechanisms. Task structures were modeled after tasks used in previous studies: Task 1 (Pahor and Jaušovec, 2018), Task 2 (Pergher et al., 2018), and Task 3 (Mohammed et al., 2017).

**Table 1:** Schematic overview of the experimental procedures, including N-Back task parameters: Stimulus type, stimulus duration, and inter-stimulus interval (ISI).

Task type	Stimulus duration	ISI	Response type	Feedback	Used stimuli
Task 1	400 ms	1,600 ms	2AFC (during ISI)	Fixation cross changes color (blue = response, red = no response)	Words pictures colors
Task 2	1,000 ms	2000 ms	Target-only (during ISI)	None (white fixation cross only)	Words pictures colors
Task 3	500 ms	2,500 ms	Target-only (during stimulus + ISI)	Visual circle (green = correct, red = incorrect)	Words pictures colors

In Task 1, stimuli were presented for 400 ms, followed by a 1,600 ms ISI. During the ISI, participants responded to both targets and non-targets using a two-alternative forced-choice design. A white fixation cross was displayed throughout the ISI, turning blue when a response was made and red if no response was made. In Task 2, stimuli were shown for 1,000 ms with a subsequent ISI of 2000 ms. During the ISI, a white fixation cross was presented, and participants were instructed to press a button only when a target was detected. No feedback was provided. In Task 3, stimuli were displayed for 500 ms, followed by a 2,500 ms ISI. Participants were instructed to respond to targets during stimulus presentation or ISI and were given feedback for correct (green circle around the stimulus) and incorrect responses (red circle around the stimulus). Of note, for Tasks 1 and 2, responses during stimulus presentation were not allowed. Importantly, the three task variants differ simultaneously on multiple task parameters; stimulus duration, ISI length, response contingency, and feedback type. Task 3 is particularly distinctive in combining the longest ISI (2,500 ms) with immediate accuracy feedback and a target-only response requirement, whereas Tasks 1 and 2 share the absence of accuracy feedback and differ primarily in stimulus duration and total trial length.

Each participant was randomly assigned to four of the nine n-back tasks, administered across two sessions conducted on different days (i.e., all four tasks were done on both days). The assignment was based on subject number to ensure an equal number of participants (*N* = 16) for each variant. Each session was structured into 11 consecutive blocks, beginning with two practice blocks (one 1-back and one 2-back practice blocks), followed by four 2-back test blocks, a 3-back practice block, and concluding with four 3-back experimental blocks. Fifteen-second rest intervals were included between blocks to minimize participant fatigue. Practice blocks consisted of 16 trials using ‘Task 3’ with color stimuli, while experimental blocks consisted of 40 + n trials (i.e., 1-back had 42 trials, 2-back had 43 trials).

Behavioral performance was logged online using Psychtoolbox (v3.0.8) and a DATAPixx stimulus unit. In line with Shalchy et al. (2020), performance was measured as behavioral accuracy (percentage of correct target responses). Behavioral accuracy values and their associated permutation-based statistical tests (30,000 iterations) were cited directly from Shalchy et al. (2020). Reaction times were omitted due to differing response contingencies across task structures.

### EEG recording

During EEG recording, participants were instructed to sit in an electrically shielded room, maintaining a relaxed posture to minimize artifact contamination in the data. The technical setup for stimulus presentation, display, and timing synchronization was consistent with that described in Shalchy et al. (2020), which included the use of MATLAB 2007b with Psychtoolbox 3.0.8 and a hardware-based timing system (a DATAPixx stimulus unit) to ensure precise synchronization of stimulus onset and response logging. Stimuli were displayed on a 22.5-inch Sony Trinitron CRT monitor with a resolution of 1,280 × 1,024 pixels and a refresh rate of 75 Hz.

EEG signals were recorded using a Biosemi ActiveTwo system with 32 active Ag/AgCl electrodes placed according to the extended 10–20 system at O1, Oz, O2, PO3, PO4, P7, P3, Pz, P4, P8, CP5, CP1, CP2, CP6, T7, C3, Cz, C4, T8, FC5, FC1, FC2, FC6, F7, F3, Fz, F4, F8, AF3, AF4, Fp1, Fp2. Six external electrodes were placed on the mastoids (two) for offline referencing and around the eyes (four) for EOG recording, capturing blinks and eye movements at the left and right canthi and above and below the left eye. Data was recorded at 2048 Hz. The EEG signal was recorded without a high-pass filter and with only hardware-level low-pass filtering applied by the recording system.

### Data preprocessing and analysis

EEG data preprocessing was performed using EEGLAB (v14.1.1; Delorme and Makeig, 2004) in MATLAB R2015a, whereas the subsequent extraction of time-frequency signals was conducted using Python 3 with the MNE tool box (Gramfort et al., 2013). The continuous EEG recordings were first downsampled from 2048 Hz to 512 Hz, a sampling rate well above the Nyquist frequency for our highest frequency of interest (30 Hz) while reducing computational demands for time-frequency analysis. A Butterworth band-pass filter was applied with cutoff frequencies set at 0.1 Hz (high-pass) and 40 Hz (low-pass) to remove slow drifts and high-frequency noise. To reduce reference bias, all electrodes were re-referenced offline to the average of the left and right mastoid electrodes (TP9 and TP10).

Following the initial preprocessing steps, the data then underwent thorough manual inspection to identify and exclude segments that contained artifacts or noise. To remove ocular artifacts such as blinks and eye movements an Independent Component Analysis (ICA) was conducted. Components reflecting these artifacts were identified visually and removed from the data. Data were then segmented into epochs around target stimuli, ranging from 1,000 ms before to 2000 ms after target onset, and baseline correction was applied by subtracting the average signal from the 200 ms pre-stimulus interval from each epoch. Epochs were rejected if the peak-to-peak voltage exceeded 100 μV within any 50 ms window, which moved through the epoch in 50 ms steps. This sliding window method allowed for the removal of brief artifacts occurring at any point within the epoch.

### Time-frequency analysis

Following preprocessing, only trials with correct responses were included in the analysis. To calculate event-related synchronization (ERS) and event-related desynchronization (ERD) patterns, power changes were computed for each epoch across all 32 channels using Morlet wavelet convolution. Time-frequency representations were computed using a Morlet wavelet with 6 cycles per wavelet, covering frequencies from 1 to 30 Hz. This number of cycles provides a balance between temporal and frequency resolution suitable for the frequency bands of interest. The 200 ms pre-stimulus period served as the baseline, against which power values were normalized using a log-ratio transform. Power was then averaged first across trials within each participant and then across participants. Increases in power within a frequency band relative to baseline indicate ERS, while decreases reflect ERD. To visualize significant ERD/ERS in the topographical maps, a bootstrap procedure was used (α = 0.01); power changes were considered significantly different from baseline when the entire 99% confidence interval was consistently above or below zero. Statistical comparisons between conditions were conducted using the procedures described in the Statistical Analysis section below.

### Statistical analysis

To investigate the spatial distribution of task-related power changes, topographical maps of theta (4–8 Hz), alpha (8–12 Hz), and beta (12–30 Hz) ERD/ERS were generated across participants for specific post-stimulus time intervals. Three regions of interest (ROIs) were defined for subsequent statistical analysis, each based on established spatial distributions of oscillatory activity: a frontal ROI for theta (Fp1, Fp2, AF3, AF4, F7, F3, Fz, F4, F8), reflecting midline frontal generators of theta ERS during working memory encoding (Cavanagh and Frank, 2014; Klimesch et al., 1997); a centro-parietal ROI for alpha (CP1, CP2, CP5, CP6, P3, Pz, P4, P7, P8), consistent with parietal alpha suppression during working memory maintenance (Jensen and Mazaheri, 2010; Wianda and Ross, 2019); and a fronto-central ROI for beta (FC1, FC2, FC5, FC6, C3, Cz, C4), reflecting the central distribution of beta modulation during cognitive control (Chen and Huang, 2016; Schmidt et al., 2019).

Mean power within the established time window of significant modulation (100–500 ms for theta, 300–700 ms for alpha, and 200–600 ms for beta) was extracted for each task structure (Task 1, Task 2, Task 3) and stimulus type (Words, Pictures, Colors). These values were averaged across the respective frequency bands and time windows. Because participants were assigned to only four of the nine task variants, the data are not fully repeated measures across all conditions. Statistical comparisons were therefore conducted using independent samples ANOVAs to examine the effects of stimulus type within each task, and the effects of task structure within each stimulus type. Post-hoc pairwise comparisons were performed using independent samples *t*-tests with false-discovery rate (FDR) corrections to control the familywise error rate.

To examine the temporal dynamics of oscillatory effects, cluster-based permutation testing was applied to the full time-resolved power series extracted from each ROI. This non-parametric approach identifies contiguous time windows (the clusters) where conditions differ reliably, while controlling for the multiple comparisons problem that arises from testing at every time point. For each comparison of interest, an F-statistic was computed at every time point and clusters of adjacent significant time points were identified. The sum of *F*-values within each cluster was then compared against a null distribution generated by randomly permuting condition labels 1,000 times. Clusters whose summed *F*-value exceeded the 95th percentile of this null distribution were considered significant (*p* < 0.05). Two types of comparisons were conducted: each condition was tested against zero to identify time windows of significant ERS or ERD relative to baseline, and conditions were compared against each other to identify time windows where task structure or stimulus type produced reliably different responses. All between-condition comparisons used independent samples tests consistent with the between-subjects design of the study. Time-course analyses were restricted to post-stimulus time points (0–2000 ms) to avoid potential edge effects from wavelet convolution in the pre-stimulus baseline period.

## Results

### Behavioral results

Behavioral accuracy (percentage of correct target responses) was reported by Shalchy et al. (2020) and summarized here. Permutation-based tests revealed a strong main effect of task structure: Task 3 yielded significantly higher accuracy than Task 1 (*p* < 0.002 across all stimulus types) and Task 2 (*p* < 0.001 for words; *p* = 0.012 for colors). Performance did not differ between Task 1 and Task 2 (*p* > 0.4). In contrast, stimulus type had no significant effect on accuracy (*p* > 0.2). These behavioral results confirm that macro-level performance was driven by task structure rather than stimulus content.

### EEG results

Time-frequency analysis of oscillatory brain activity during n-back task performance revealed distinct patterns of frequency-specific modulation as a function of both task structure and stimulus type. To verify the spatial distribution of these effects and validate the theoretically motivated ROI sets of electrodes, we first generated topographical maps of theta (4–8 Hz), alpha (8–12 Hz), and beta (12–30 Hz) ERD/ERS for each of the nine task variants (Figure 1). Visual inspection of these topomaps shows that theta synchronization has a predominantly frontal distribution, most clearly for word and picture stimuli, while alpha desynchronization shows a clear and stable posterior centro-parietal distribution regardless of task or stimulus type. Beta shows no real consistent spatial pattern. This topographical distribution is broadly consistent with the theoretically defined ROIs used in subsequent statistical analyses, with the frontal theta and centro-parietal alpha patterns aligning well with established spatial signatures of these frequency bands during working memory performance.

**Figure 1:**
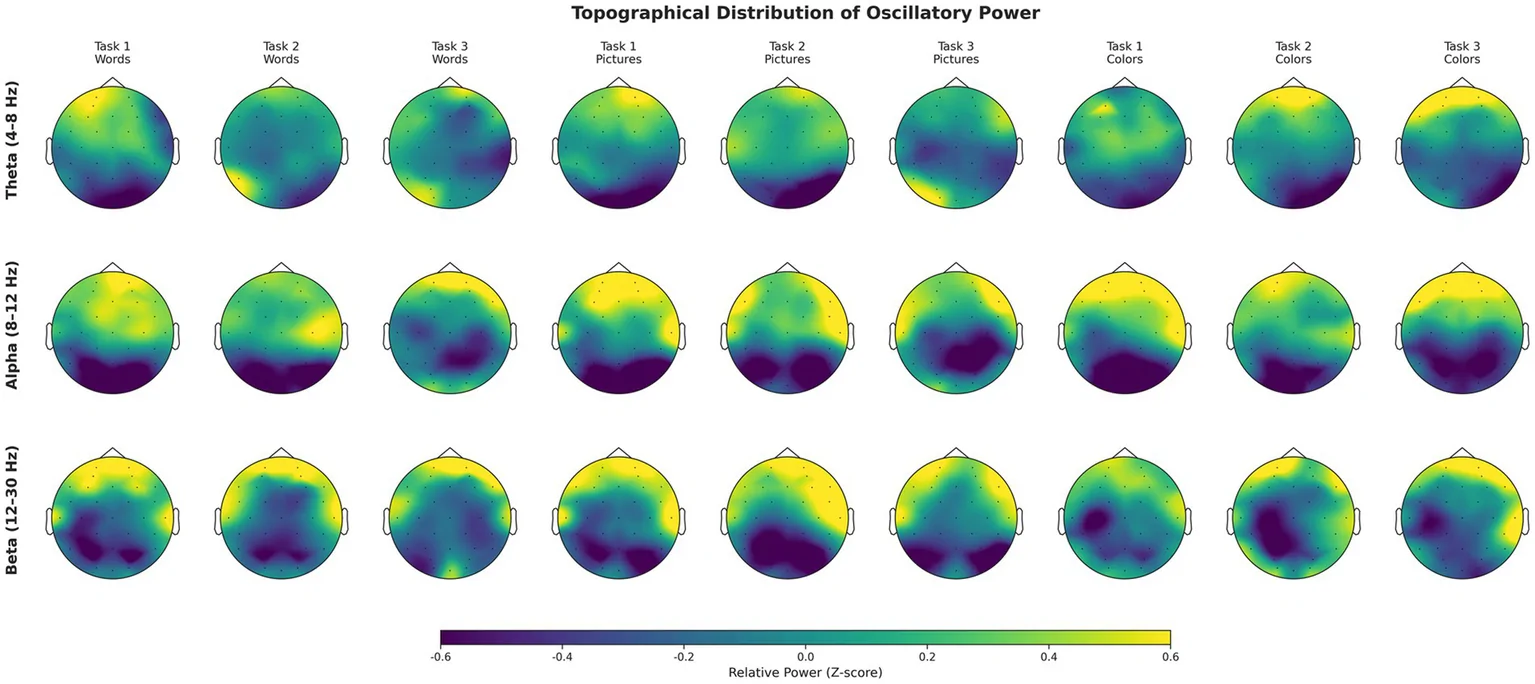
Topographical distribution of oscillatory power across N-back task variants. Grand-average topographical maps showing scalp distribution of oscillatory power changes in theta (4–8 Hz, 100–500 ms; top row), alpha (8–12 Hz, 300–700 ms; middle row), and beta (12–30 Hz, 200–600 ms; bottom row) across all nine task variants. Color values reflect z-scored power relative to the spatial mean, with yellow-green indicating power increases (ERS) and dark blue indicating power decreases (ERD). Theta synchronization is observed in frontal regions; alpha desynchronization is consistently posterior; beta shows no stable spatial pattern.

### Stimulus-driven effects: frontal theta synchronization

Figure 2 shows the stimulus driven effects at each of the three frequency bands, separately for each task as derived from the theoretically motivated ROIs and time course. Results showed that frontal theta synchronization (4–8 Hz) was significantly modulated by stimulus type (*F*(2,105) = 6.58, *p* = 0.0019), whereas task structure had no significant effect on theta power (*F*(2,105) = 2.00, *p* = 0.138). This pattern confirms the first half of the predicted dissociation: theta indexes what stimuli are being encoded, not how the task is structured.

**Figure 2:**
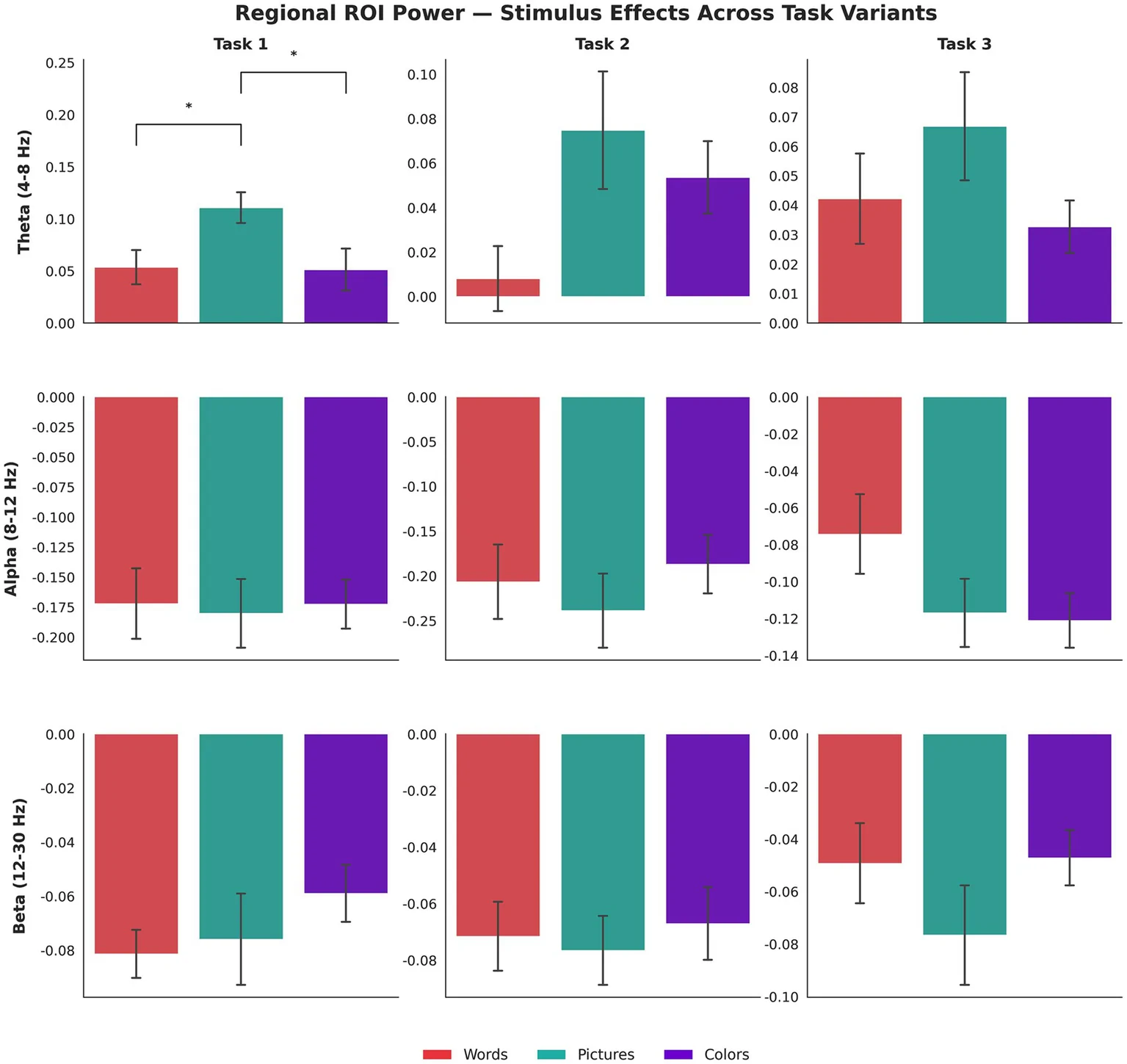
Oscillatory power as a function of stimulus type. Mean power (log ratio relative to baseline) extracted from band-specific ROIs for each stimulus type (Words, Pictures, Colors) separately for each task variant. Rows show theta (4–8 Hz, frontal ROI; top), alpha (8–12 Hz, centro-parietal ROI; middle), and beta (12–30 Hz, fronto-central ROI; bottom). Positive values reflect ERS, negative values reflect ERD. Significant differences between stimulus types are marked with asterisks (*p* < 0.05, FDR corrected). Error bars represent ±1 SEM.

However, upon further inspection of each task revealed that the stimulus effect was driven predominantly by Task 1, the shortest and most time-pressured of the three variants. Indeed, Task 1 showed a significant effect of stimulus type (*F*(2,33) = 3.78, *p* = 0.0296), with picture stimuli eliciting significantly greater theta synchronization than both words (*p* = 0.035) and colors (*p* = 0.035). Task two showed a near-significant effect of stimulus type (*F*(2,33) = 2.92, *p* = 0.0619) with the largest synchronization effect observed for picture stimuli. Task 3 (*F*(2,33) = 1.43, *p* = 0.2510) showed no significant stimulus effects at all. The disappearance of this effect across task variants is consistent with the view that stimulus-driven encoding demands are most pronounced in Task 1, which uniquely combines the shortest total trial duration with a requirement to respond to all stimuli rather than targets only. However, given that the three tasks differ simultaneously on multiple structural dimensions including stimulus duration, ISI length, response contingency, and feedback type, the specific parameter driving this pattern cannot be determined from the current data alone. Crucially, theta power showed no significant task structure effects for any stimulus type (Words: *p* = 0.108, Pictures: *p* = 0.279, Colors: *p* = 0.594), confirming that frontal theta is selectively sensitive to stimulus content and not to the broader procedural parameters of the task. Importantly, no effect of Stimulus Type was observed for the alpha band (*F*(2,105) = 0.63, *p* = 0.5340); with no significant stimulus effects observed within any individual task (all *p* > 0.15) supporting the hypothesis that theta, not alpha encodes stimulus type. Similarly, no effect of stimulus type was observed in the beta band (*p* = 0.237).

### Task-structure related effects: centro-parietal alpha desynchronization

Unlike stimulus type, task structure produced a highly significant effect on alpha power (*F*(2,105) = 13.94, *p* < 0.0001; see Figure 3), further confirming the dissociation: while theta tracked stimulus content, alpha tracked how the task was structured. Neither band appeared to track the other variable.

**Figure 3:**
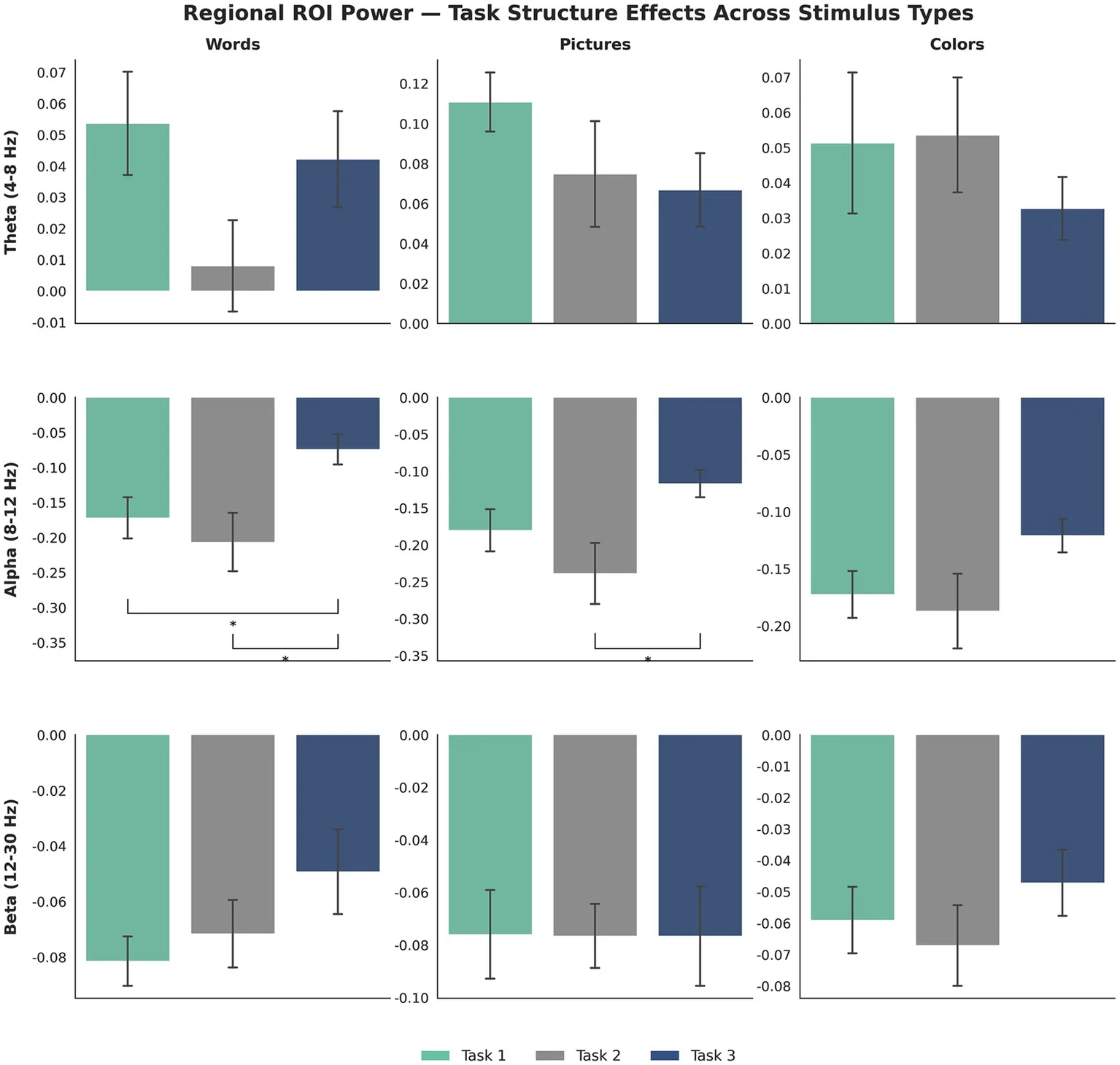
Oscillatory power as a function of task structure. Mean power (log ratio relative to baseline) extracted from band-specific ROIs for each task variant (Task 1, Task 2, Task 3) separately for each stimulus type (Words, Pictures, Colors). Rows show theta (4–8 Hz, frontal ROI; top), alpha (8–12 Hz, centro-parietal ROI; middle), and beta (12–30 Hz, fronto-central ROI; bottom). Positive values reflect ERS, negative values reflect ERD. Significant differences between task variants are marked with asterisks (* *p* < 0.05, FDR corrected). Error bars represent ±1 SEM.

Post-hoc testing showed that the task structure effect was most consistent for Words and Pictures. For Words, alpha suppression differed significantly across task variants (*F*(2,33) = 4.69, *p* = 0.0149), with Task 3 showing significantly less suppression than both Task 1 (*p* = 0.017) and Task 2 (*p* = 0.017), while Tasks 1 and 2 did not differ from each other (*p* = 0.503). A similar pattern emerged for Pictures (*F*(2,33) = 3.82, *p* = 0.0275), where Task 2 showed significantly greater suppression than Task 3 (*p* = 0.035). Colors showed no significant task structure effects (*F*(2,33) = 2.13, *p* = 0.1316).

The finding that Task 3 consistently shows the least alpha suppression is consistent with the view that its combination of the longest ISI (2,500 ms), immediate accuracy feedback, and target-only responding reduces the need for sustained active maintenance and suppression of competing information across the inter-trial interval. However, given that Task 3 differs from Tasks 1 and 2 on multiple structural dimensions simultaneously, the specific parameter responsible for this pattern cannot be definitively extracted from the current data.

### Beta-band stability

Beta-band oscillations (12–30 Hz) showed no significant modulation by either stimulus type or task structure at the level of mean ROI power. The global ANOVA revealed no significant effect of stimulus type (*F*(2,105) = 1.45, *p* = 0.2374) or task structure (*F*(2,105) = 1.12, *p* = 0.3267), and no significant comparisons emerged for any stimulus type or task variant combination (all *p* > 0.18).

If nothing else, this overall beta stability serves as indirect evidence that the selective modulation observed in theta and alpha cannot be attributed to some non-specific global shift in cortical excitability. If that were the case beta would most likely show this effect as well. Instead, the observed null result provides a measure of validity for the dissociation: the brain is not simply modulating its overall activity in response to task demands, but rather recruits specific frequency-band mechanisms for specific cognitive functions.

### Stimulus-driven temporal dynamics

To examine the precise time course of the stimulus-driven oscillatory effects, we plotted the fluctuations in power over time and compared the resulting traces across stimulus types within each task variant (Figure 4; Table 2, Panel A). For frontal theta (4–8 Hz), picture stimuli elicited a visibly larger synchronization peak in Task 1 compared to words and colors, emerging rapidly around 150 ms post-stimulus and peaking between 250 and 400 ms. Cluster-based permutation testing confirmed a single significant between-stimulus cluster in the entire dataset: words versus pictures in Task 1 (201–717 ms post stimulus onset). No equivalent significant clusters were present in Tasks 2 or 3. This early stimulus-driven theta effect is consistent with the ROI findings and suggests a more precise timecourse: it is a fast, encoding-related response confined to the first 500 ms post-stimulus. Beyond this window, all three stimulus lines converge regardless of task variant, suggesting that stimulus-driven differences in encoding demand are resolved early and do not persist into later maintenance-related processing.

**Figure 4:**
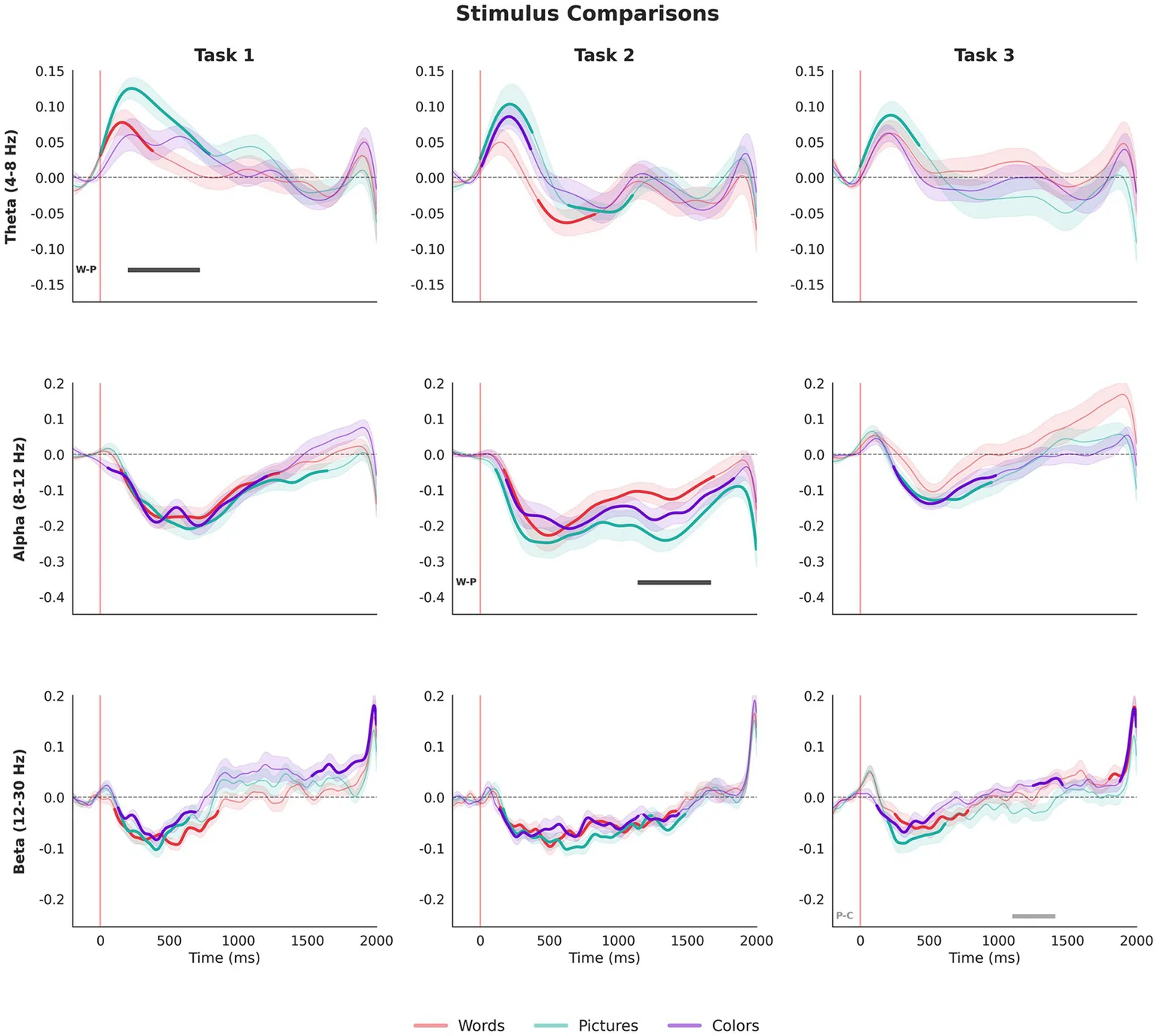
Time-resolved stimulus comparisons across task variants. Grand-average time courses of oscillatory power (log ratio relative to baseline) for each frequency band (rows: Theta 4–8 Hz, alpha 8–12 Hz, beta 12–30 Hz) and task variant (columns: Task 1, Task 2, Task 3). Words (blue), pictures (orange), colors (purple). Shaded regions reflect ±1 SEM. Thick line segments indicate time windows of significant deviation from baseline (cluster permutation, *p* < 0.05). Horizontal bars below each panel mark time windows where pairs of stimulus types differed significantly from each other. Vertical red line marks stimulus onset; horizontal dashed line marks baseline.

**Table 2:** Significant cluster timings for Figures 4, 5.

A. Stimulus comparisons			
Band	Task	Comparison	Time window (ms)
Theta (4–8 Hz)	Task 1	Words vs. Pictures	201–717
Alpha (8–12 Hz)	Task 2	Words vs. Pictures	1,141–1,666
Beta (12–30 Hz)	Task 3	Pictures vs. Colors	1,104–1,408

B. Task comparisons			
Band	Stimulus	Comparison	Time window (ms)
Theta (4–8 Hz)	Words	Task 1 vs. Task 2	336–770
Theta (4–8 Hz)	Pictures	Task 1 vs. Task 2	443–1,172
Theta (4–8 Hz)	Colors	Task 1 vs. Task 2	475–930
Alpha (8–12 Hz)	Words	Task 1 vs. Task 3	51–465, 566–1,043, 1,506–2000
Alpha (8–12 Hz)	Words	Task 2 vs. Task 3	18–2000
Alpha (8–12 Hz)	Pictures	Task 1 vs. Task 2	1,074–2000
Alpha (8–12 Hz)	Pictures	Task 2 vs. Task 3	0–652, 955–2000
Alpha (8–12 Hz)	Colors	Task 1 vs. Task 2	1,148–1936
Alpha (8–12 Hz)	Colors	Task 1 vs. Task 3	6–441
Alpha (8–12 Hz)	Colors	Task 2 vs. Task 3	600–1902
Beta (12–30 Hz)	Words	Task 1 vs. Task 2	881–1,232
Beta (12–30 Hz)	Words	Task 2 vs. Task 3	875–1,232, 1,262–1,547
Beta (12–30 Hz)	Pictures	Task 1 vs. Task 2	748–1,238, 1,258–1,502
Beta (12–30 Hz)	Colors	Task 1 vs. Task 2	809–1,643
Beta (12–30 Hz)	Colors	Task 2 vs. Task 3	592–756, 812–1,545

That ‘2000 ms’ post-stimulus serves as the end of the epoch. Theoretically the effect could persist beyond this cut-off.

For centro-parietal alpha (8–12 Hz), stimulus type traces were largely overlapping across all three task variants in the early post-stimulus window, with no significant between-stimulus clusters emerging before 500 ms in any task. This early null coincides with the time window where theta shows its strongest stimulus sensitivity, once more confirming that the two bands are modulated by different information during this critical encoding period. A single late significant cluster was observed for words versus pictures in Task 2, spanning 1,141–1,666 ms, suggesting that some stimulus sensitivity in alpha emerges later in the processing epoch. This late effect falls outside the 300–700 ms time window used for ROI extraction and therefore does not easily compare to the primary analysis, but it suggests that some stimulus sensitivity in alpha may emerge in later processing stages outside our theoretically defined time window.

For fronto-central beta (12–30 Hz), stimulus type traces were largely overlapping across all three task variants throughout the epoch, with only a late and isolated cluster for pictures versus colors in Task 3, spanning 1,104–1,408 ms. This cluster falls well outside the theoretical 200–600 ms time window used for beta ROI extraction and may well reflect late motor or response-related activity rather than a meaningful stimulus-driven effect on working memory processes.

To examine the precise time course of the oscillatory effects related to task structure, we plotted the fluctuations in power over time and compared the resulting traces across tasks separately for each stimulus type (Figure 5; Table 2, Panel B). Results revealed a complementary but temporally more complex picture.

**Figure 5:**
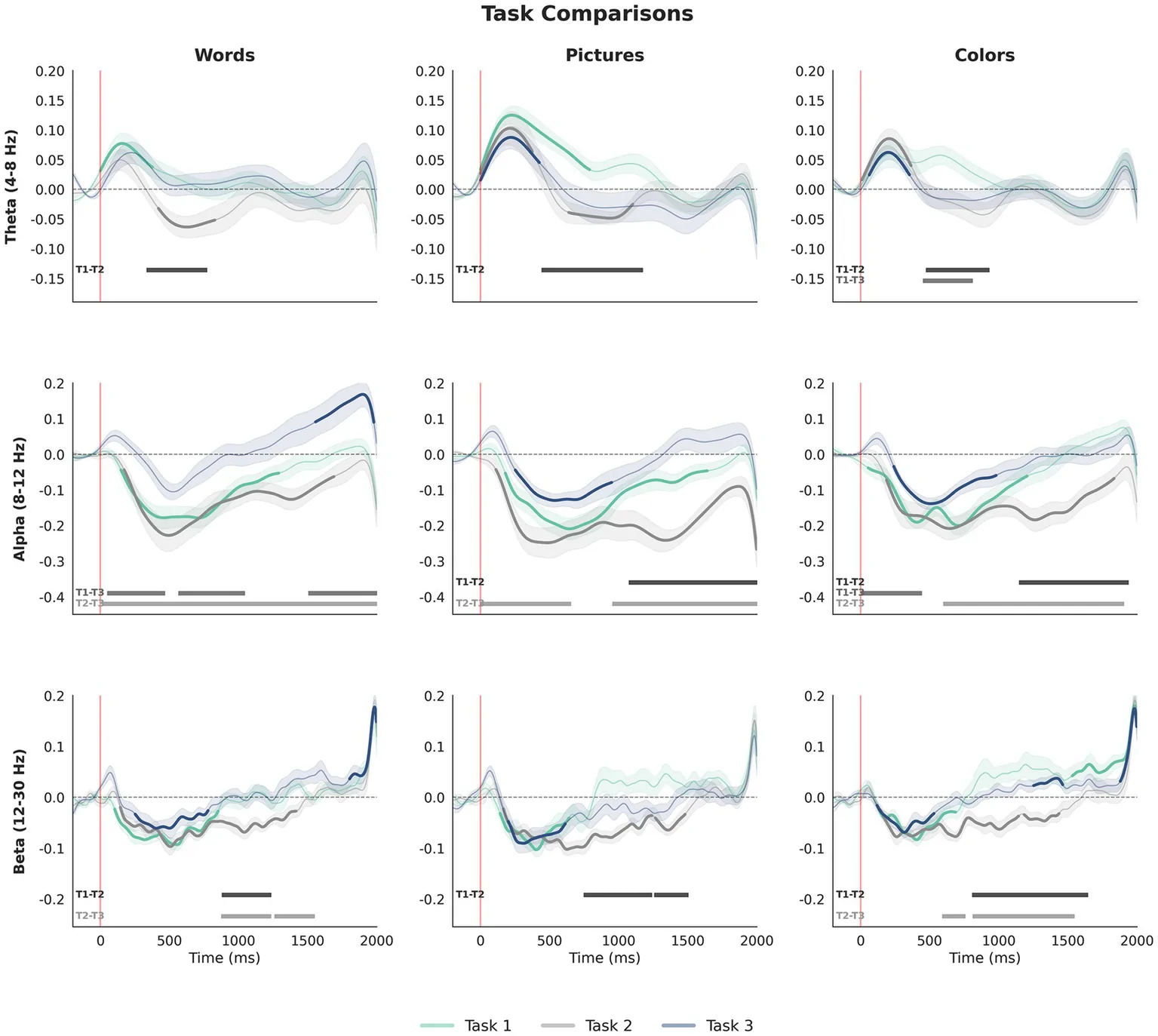
Time-resolved task structure comparisons across stimulus types. Grand-average time courses of oscillatory power (log ratio relative to baseline) for each frequency band (rows: Theta 4–8 Hz, Alpha 8–12 Hz, Beta 12–30 Hz) and stimulus type (columns: Words, Pictures, Colors). Task 1 (green), Task 2 (grey), Task 3 (dark blue). Shaded regions reflect ±1 SEM. Thick line segments indicate time windows of significant deviation from baseline (cluster permutation, *p* < 0.05). Horizontal bars below each panel mark time windows where pairs of task variants differed significantly from each other. Vertical red line marks stimulus onset; horizontal dashed line marks baseline.

For alpha (8–12 Hz), clear and sustained differentiation between task variants emerged around 200–300 ms post-stimulus and persisted well beyond 1,000 ms across all three stimulus types. Cluster-based permutation testing confirmed significant between-task clusters consistently showing Task 3 producing less alpha suppression than Tasks 1 and 2 throughout much of the epoch. This sustained effect is mostly consistent with the ROI findings and suggests that unlike the early and transient theta stimulus effect, the task structure effect on alpha is a slower and more persistent modulation that reflects sustained differences in cortical engagement across the full processing window rather than a rapid encoding-related response.

For theta (4–8 Hz), task-based power modulations largely overlapped in the early 150–400 ms window where stimulus effects were most prominent, consistent with the ROI null result for task structure. However, significant between-task clusters emerged beyond 300 ms across all three stimulus types, driven primarily by Task 1 versus Task 2 differences (Words: 336–770 ms, Pictures: 443–1,172 ms, Colors: 475–930 ms), indicating that task structure does exert a meaningful influence on theta, but only after the early stimulus-driven encoding response has resolved. This temporal pattern suggests that frontal theta is recruited first for stimulus encoding, and that task-structural differences in cognitive processing emerge only in the later phase of the response when encoding demands have been met and maintenance-related processes take over.

For beta (12–30 Hz), late between-task clusters were observed in the 750–2000 ms window across stimulus types, particularly for T1-T2 comparisons. These likely reflect differences in motor preparation and response execution across task variants rather than working memory processes per se, consistent with the established role of beta in motor and premotor processing (Schmidt et al., 2019).

Taken together, results suggest that the functional dissociation between stimulus-driven theta and task-driven alpha is most pronounced in the early post-stimulus window. During the first 300 ms, theta selectively differentiates stimulus types while alpha remains insensitive to stimulus content; a clean temporal dissociation that maps directly onto the ROI findings. Beyond this early window the picture becomes more complex: alpha shows some late stimulus sensitivity and theta shows task structure effects, suggesting that the two bands become less functionally distinct as processing moves from initial encoding into sustained maintenance. This temporal elaboration does not diminish the core ROI-based finding that stimulus type is the dominant driver of theta and task structure is the dominant driver of alpha. However, it adds an important detail: the dissociation is strongest, and perhaps most functionally meaningful, in the early encoding phase of working memory processing.

## Discussion

This study investigated task-related oscillatory brain activity during performance of n-back working memory tasks, focusing on time-frequency analysis of ERD/ERS changes across nine systematically varied n-back task variants. The findings provide evidence for a functional double dissociation: frontal theta activity is selectively sensitive to stimulus type, suggesting that it reflects stimulus encoding processes. Centro-parietal alpha activity is selectively sensitive to task structure, reflecting the temporal and procedural context in which encoding occurs. Beta oscillations showed minimal responsiveness to either manipulation at the level of mean ROI power, serving as a functional control for the specificity of the theta and alpha effects and appears to suggest the encoding of domain general processes. Together, these findings demonstrate that relatively simple design choices in n-back tasks can yield systematically different neural signatures depending on which frequency band is examined, with important implications for how EEG-based cognitive assessments are designed and interpreted.

Frontal theta synchronization was significantly modulated by stimulus type, with picture stimuli eliciting greater synchronization than words and colors but this only occurred when stimulus durations were relatively short (400 ms; Task 1). An increase in oscillatory activity within the theta frequency range over frontal brain regions has been reported as a likely neural correlate of the central executive monitoring component fundamental to all working memory models (Berger et al., 2014; Cohen, 2011). More specifically, frontal theta oscillations may signal the need for greater allocation of attentional resources and cognitive effort during task performance (Sauseng et al., 2010; Scharinger et al., 2017). The fact that pictures drive stronger theta predominantly in Task 1 may suggest that the unique properties of this specific task enhance differential frontal encoding demands across stimulus types. Task 1 is distinctive in requiring responses to all stimuli rather than targets only, combined with the shortest total trial duration. Such task properties may increase sustained attentional demands across all trials regardless of stimulus identity, thereby making the frontal system more sensitive to differences in stimulus content. Importantly, this theta modulation was driven specifically by picture stimuli relative to both words and colors, which did not differ from each other. Rather than reflecting a broad distinction between multimodal (visual + verbal) and unimodal/verbal encoding—which would predict similar elevations for pictures and colors—this pattern suggests that pictures uniquely demand greater frontal executive recruitment during rapid encoding. Unlike simple colors, which are easily recoded into single verbal labels, or printed words, complex pictures engage both high visual feature processing and automatic semantic dual-coding (Paivio, 1971), placing higher immediate demands on the frontal central executive under time-pressured presentation. This interpretation is in line with the view that theta synchronization reflects online allocation of attentional resources under cognitive demand (Cavanagh and Frank, 2014) but also shows that the sensitivity of theta to stimulus content is contingent on the temporal parameters of the task. Crucially, theta activity showed no significant task structure effects for any stimulus type, confirming that frontal theta is selectively tuned to stimulus content rather than to the broader procedural parameters of how the task is organized.

ROI-based alpha-band desynchronization showed a complementary and contrasting pattern. Centro-parietal alpha suppression was not modulated by stimulus type across all tasks, while being strongly and significantly modulated by task structure. Alpha power typically shows desynchronization in centro-parietal regions due to attention-related processes and the inhibition of task-irrelevant information (Missonnier et al., 2013; Wianda and Ross, 2019). The observed reduced alpha suppression in Task 3 is consistent with its longest ISI and immediate accuracy feedback reducing sustained maintenance demands across the inter-trial interval. When uncertainty about performance is resolved immediately and more time separates successive stimuli, the brain may need to recruit fewer inhibitory resources. Tasks 1 and 2, which share the absence of accuracy feedback and require sustained monitoring across shorter inter-trial intervals, show correspondingly greater alpha suppression. However, as with the theta findings, the somewhat confounded nature of the various task parameters means that this interpretation needs to be interpreted tentatively and future work should systematically isolate the contribution of each structural feature. The absence of any stimulus type effect on ROI-based alpha completes the double dissociation and confirms that centro-parietal alpha tracks the structural demands of the task context rather than the identity of the stimuli being processed.

The cluster permutation time course analyses add important temporal information to these findings. For theta, while the mean ROI analysis confirmed stimulus sensitivity only in Task 1, the permutation tests revealed subtle but significant temporal clusters distinguishing task variants in the 400–600 ms window. These later theta differences appear to suggest that theta is sensitive to task structure, but only at later processing stages when stimulus encoding has already been conducted. For alpha, the permutation tests revealed sustained between-task clusters persisting well beyond 1,000 ms, once more suggesting that task structure effects on alpha reflect a sustained difference in cortical engagement rather than a brief transient response. The late beta clusters observed in the permutation analysis, particularly for T1-T2 comparisons, likely reflect differences in motor preparation and response execution across task variants rather than working memory-related processes per se, consistent with the established role of beta in motor and premotor processing (Schmidt et al., 2019).

Nonetheless, beta oscillations, which have recently been linked to cognitive processes including working memory, showed no significant modulation by stimulus type or task structure at the level of mean ROI power. This relative stability confirms that the selective modulation observed in theta and alpha does not reflect a non-specific global shift in oscillatory activity, but rather functionally distinct responses tied specifically to encoding and maintenance demands.

The current findings extend the earlier ERP-based investigation of this dataset by Shalchy et al. (2020) by transitioning from phase-locked neural responses to a frequency-specific interrogation of working memory processes. While Shalchy and colleagues demonstrated that early sensory-perceptual components (N1, P2) and later post-sensory processing, cognitive markers (P3) are modulated by task variants, the current time-frequency analysis reveals that these effects are underpinned by a functional dissociation between oscillatory systems that ERPs cannot fully capture. The sensitivity of frontal theta synchronization to stimulus content, and in particular for more complex visual stimuli in high-pressure encoding environments, largely parallels the stimulus-driven shifts in N1/P2 morphology reported in the ERP study. However, our results suggest that theta oscillations provide a more specific index of the allocation of attentional resources required for encoding, especially when temporal windows are short. Similarly, the observed modulation of alpha activity by task structure dovetails the ERP evidence regarding late-stage P3 differences across task variants. While the P3 reflects discrete stages of stimulus evaluation and categorization, the sustained alpha suppression observed here appears to track the broader neural demand for inhibitory activity and the maintenance of the task set.

The finding that Task 3 consistently elicits qualitatively different neural signatures in both ERP and oscillatory domains, characterized by enhanced performance and reduced neural suppression, suggests that its specific structural design may facilitate recruitment of cognitive resources. Mechanistically, our work shows that while ERPs effectively index the timing of specific processing stages, the dissociation between theta and alpha power changes provides a more comprehensive account of how stimulus identity and task parameters independently shape the neural cost of working memory maintenance.

These findings have direct implications for researchers designing n-back protocols and interpreting neural biomarkers. Studies examining frontal theta as a biomarker of executive function, for instance in ADHD or dementia populations, should carefully consider stimulus type and ensure it is matched across groups, as stimulus type and complexity drive theta selectively depending on the structural properties of the task. Investigations targeting parietal alpha suppression as a capacity marker should prioritize control of timing parameters, as task structure is the primary driver of alpha modulation. Crucially, failing to account for these task-dependent oscillatory modulations could lead to misinterpretations of the underlying cognitive processes, particularly in clinical assessments. For example, if a clinical study compares a standard continuous n-back task against a variant of the n-back task that uses explicit cues, structured intervals, or immediate feedback (such as Task 3), the observed reduction in alpha suppression or altered frontal theta dynamics might be mistakenly interpreted as a structural impairment in working memory capacity or neural gating. Nevertheless, the group difference may simply reflect task-driven shifts in cognitive strategy. More broadly, these findings demonstrate that the choice of n-back task variant is not methodologically neutral with respect to EEG outcomes: different variants clearly emphasize different neural systems, and comparisons across studies using different task designs should account for this systematically.

One potential consideration in n-back paradigms is the role of working memory load (e.g., 2-back vs. 3-back). In the present study, data were collapsed across 2-back and 3-back test blocks to maximize trial counts and maintain high signal-to-noise ratios for time-frequency power estimation and cluster-based permutation analyses across our between-subject task groupings. While higher working memory load typically elevates overall frontal theta synchronization and centro-parietal alpha suppression (e.g., Scharinger et al., 2017), our primary focus was determining whether stimulus identity and task structure recruit fundamentally distinct frequency bands regardless of memory load. Collapsing across load levels ensured robust statistical power to isolate these primary task-parameter effects. Nevertheless, future studies designed with higher trial numbers per condition could explicitly examine whether working memory load interacts with task structure; for example, testing whether heavy load magnifies alpha suppression differences under time-pressured task variants.

Finally, while the current study focused on theta, alpha, and beta bands, growing evidence suggests that high-frequency gamma-band oscillations (>30 Hz) also play a crucial role in working memory, particularly in item maintenance and phase-amplitude coupling with lower-frequency rhythms (Lundqvist et al., 2018). Due to our preprocessing low-pass filter cutoff (40 Hz) designed to minimize muscle artifact contamination, we chose not to investigate gamma-band dynamics. Future investigations using advanced artifact-rejection techniques should incorporate gamma-band activity to explore how task parameters influence high-frequency local processing alongside the theta and alpha (and beta) networks examined here.

To summarize, this study demonstrates a functional double dissociation in how neural oscillations respond to n-back task parameters: frontal theta is sensitive to stimulus type while centro-parietal alpha is sensitive to task structure, with beta showing no systematic modulation of either at the ROI level. The convergence of ROI-based and time-resolved analyses shows that the dissociation holds not only in terms of overall magnitude but also in its temporal signature. Additionally, we acknowledge our sample was overly representative of women, and therefore the generalizability of our results may be limited given recent research showcases sex-related differences in working memory neural dynamics (e.g., Hill et al., 2014). Future work should replicate our findings across larger and balanced samples, as well as extend the investigation to other task parameters and clinical populations. Critically, the three task variants examined here differ simultaneously on multiple structural dimensions including stimulus duration, ISI length, response contingency, and feedback type. While overall informative, this limits our ability to attribute the observed neural dissociations to any single parameter. Designs that systematically isolate each of these features independently will be essential for determining which specific aspect of task structure drives the alpha modulation and which aspect modulates the temporal boundary of the theta stimulus effect.

## Data Availability

Publicly available datasets were analyzed in this study. The used dataset was inherited from Shalchy et al. (2020). This data can be found at: https://www.frontiersin.org/journals/human-neuroscience/articles/10.3389/fnhum.2020.549966/full#supplementaryMaterial.
